# Nonlinear Vibration of a Micro Piezoelectric Precision Drive System

**DOI:** 10.3390/mi10030159

**Published:** 2019-02-26

**Authors:** Chong Li, Wei Zhong, Jiwen Fang, Lining Sun

**Affiliations:** 1 School of Mechanical Engineering, Jiangsu University of Science and Technology, Zhenjiang 212003, China; zhongwei@just.edu.cn (W.Z.); fjw617@just.edu.cn (J.F.); 2 Robotics and Microsystems Center, Scoochow University, Suzhou 215021, China; lnsun@hit.edu.cn

**Keywords:** nonlinear vibration, drive system, piezoelectric actuated, dynamic performance

## Abstract

A micro piezoelectric precision drive system is proposed, which is advantageous due its small size, large transmission ratio, and large output torque. The working principle of the proposed piezoelectric precision drive system is presented, and the nonlinear dynamic model and equations of the system are established. Using the Linz Ted-Poincaré and perturbation methods, the nonlinear approximate solutions of the dynamic equations are calculated. The results indicate that the nonlinear intensity of the drive system is inversely proportional to the number of meshing movable teeth. It was also noted that the rotor is most affected by the nonlinear phenomenon. These results can be utilized both to optimize the dimensions of the piezoelectric precision drive system and to reduce the intensity of vibrations during operation.

## 1. Introduction

With the development of science and technology, the precision transmission has been widely utilized in society. At the same time, numerous novel transmissions such as the contactless air conveyor system, magnetic coupling transmission, and piezoelectric drive system have been developed [1,2,3,4]. Among them, the piezoelectric drive system is attracting increasing attention from scholars.

The piezoelectric actuator has superior performances, such as a fast response, a large output force, a small size, and no electromagnetic interference. As a result, it has been successfully used in the fields of precision positioning, robot actuating, energy harvesting, and vibration reduction [5,6,7,8].

Numerous developments in piezoelectric driving have been achieved. Ozaki et al. [9] proposed a bioinspired flapping-wing micro aerial vehicle with piezoelectric direct-driven actuation, and a two-wing prototype with a wingspan of 114 mm was designed and fabricated. The total mass of the aerial vehicle was 598 mg, and the maximum measured lift force was 6.52 mN at a driving voltage of 100 V. Utilizing a piezoelectric bimorph, David et al. [10] developed an improved tactile sensor. As the sensor can distinguish soft materials, it was used for brain tumor resection. Besides, Zhang et al. [11] designed a finger joint based on a hybrid multi-degree of freedom (DOF) piezoelectric ultrasonic motor. The stator of the piezoelectric motor consisted of a multi-layered piezoelectric longitudinal vibrator and a sandwich bending vibrator, which could generate a high longitudinal vibration velocity at a low input voltage. Through a testing experiment, a maximum torque of 23.5 mNm was obtained. Tajitsu [12] designed a piezoelectric poly-L-lactic acid fabric. The fabric could feel people’s movement. Thus, it was used for controlling a humanoid robot. Moreover, a novel piezoelectric-driven robot was proposed by Dharmawan et al. [13]. The robot consisted of a piezoelectric unimorph actuator and a four-bar linkage. Driven by the piezoelectric force, two four-bar linkages generated forward and backward movements. Fang et al. [14] proposed a precision position stage with a piezoelectric actuator. In addition, Zou et al. [15] developed an insect-scale flapping-wing robot with a wingspan of 35 mm. The robot had piezoelectric actuators. Li [16] proposed a novel piezoelectric-based unmanned underwater vehicle with the functions of piezoelectric vibration reduction and piezoelectric energy harvesting.

Regarding the nonlinear dynamic characteristic of the piezoelectric system, numerous works have also been conducted. Considering the inertial, geometric, and piezoelectric nonlinearities, Abdelkefi et al. [17] presented a nonlinear calculating method for an energy harvester. Using the Galerkin technique, Gauss law, and extended Hamilton principle, a reduced-order model of the harvester was built. The effect of nonlinear piezoelectric coefficients on the system response was also investigated. Moreover, Meesala et al. [18] developed a method of multiple scales as an approximate solution, as well as amplitude and phase modulation equations, to capture the nonlinear behavior of an energy harvester. With this method, the parameters of the nonlinear piezoelectric constitutive relations were estimated. Takahashi et al. [19] studied the nonlinear behavior of the ferroelectric ceramic, which excited at a resonant mode. It was found that an induced vibration stress had a greater effect on the nonlinearity than an electric field. In addition, Ozaki et al. [20] studied nonlinear piezoelectric vibration in resonant piezoelectric devices. The purpose of the study was to introduce a nonlinear parameter to the finite elements method (FEM) and to establish a method for measuring the nonlinear parameter by evaluating a nonlinear model for the piezoelectric vibration. The abovementioned studies have focused on the nonlinear vibration of the piezoelectric material itself. However, the effects of the piezoelectric nonlinear characteristic on the driving system have had fewer studies.

In this study, a micro piezoelectric precision drive system is proposed. The system integrates piezoelectric driving and a movable tooth transmission, and it has the advantages of being small in size, with a large transmission ratio, and a large output torque. Compared with the electromagnetic drive, it has merits such as a compact structure, a fast response, electromagnetic interference resistance. The nonlinear dynamic performance results in a drive system with a deteriorated load-carrying capability. For the proposed piezoelectric precision drive system, the nonlinear free vibration of the transmission part [21] and the nonlinear dynamics of the driving part under piezoelectric excitation [22] are investigated. Meanwhile, using the numerical method, the chaotic vibrations of the driving part, transmission part, and nonlinear axis force are analyzed separately [23,24,25]. In [24], the chaotic vibration of a movable tooth system was a special nonlinear vibration, but emphasis was placed on the chaotic characteristic and the influence of parameters on chaotic vibrations. However, the nonlinear response characteristic of a movable tooth transmission under piezoelectric excitation was not investigated. Unlike previous works, this new research mainly focuses on the nonlinear solution process, the influence law of parameters on the frequency characteristic, and force vibration rules, which are the different from the previous chaotic characteristics.

The aim of this study is to reveal the nonlinear vibration response law under the nonlinear piezoelectric effect. Using the dynamic theory of a discrete system, the nonlinear dynamic model of a robot-used piezoelectric precision drive system is built. With the Linz Ted-Poincaré and perturbation methods, the approximate nonlinear dynamic responses are solved. Furthermore, the effects of the system parameters on the nonlinear natural characteristic and dynamic responses are analyzed. These results can be used to reduce the vibration of the proposed drive system.

## 2. Operating Principle of the Drive System

**Figure 1:**
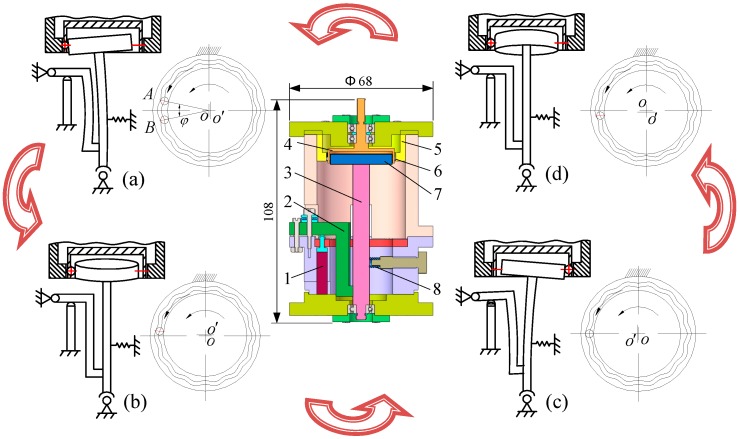
Operating principle of the micro piezoelectric precision drive system. (**a**) Initial position and 360° position; (**b**) 90° position; (**c**) 180° position; (**d**) 270° position.

The operating principle of the proposed micro piezoelectric precision drive system is shown in Figure 1. It consists of the following parts: Piezoelectric actuator (1), displacement magnifying mechanism (2), swaying rod (3), rotor (4), central gear (5), movable tooth (6), harmonic generator (7) and preload spring (8). The system is driven by two piezoelectric actuators, with dimensions of 5 mm × 5 mm × 30 mm, which are installed at the lower shell, with a phase difference of 90°. 

In the initial state, the two piezoelectric actuators maintain the original length, the harmonic rod contacts tightly with the movable teeth under the force of the preloaded spring, and the swaying rod sways toward the displacement magnifying mechanism - the random movable tooth located at the A-site in Figure 1a.

When the proposed piezoelectric precision drive system works, sinusoidal signals with a 90° phase difference are applied to the two piezoelectric actuators. Then, the actuators are lengthened in their axis direction. Driven by the deformation force, a harmonic wave is generated under the displacement amplification of the swaying rod. The harmonic force of the harmonic generator pushes the movable teeth slide along the central gear profile. 

When the swaying rod sways to the 90° position, the movable tooth moves into the middle position of the adjacent addendum and root (see Figure 1b) and then moves into the adjacent roots at the 180° position (see Figure 1c). Similarly, the swaying rod sways to the 360° position and returns to the initial position, the movable tooth moves into B-site of the adjacent addendum in Figure 1a, and it accomplishes the *φ* angle movement of a period. Thus, driven by the continuous input signal, a continuous rotation of the rotor can be generated.

## 3. Dynamic Models and Equations

**Figure 2:**
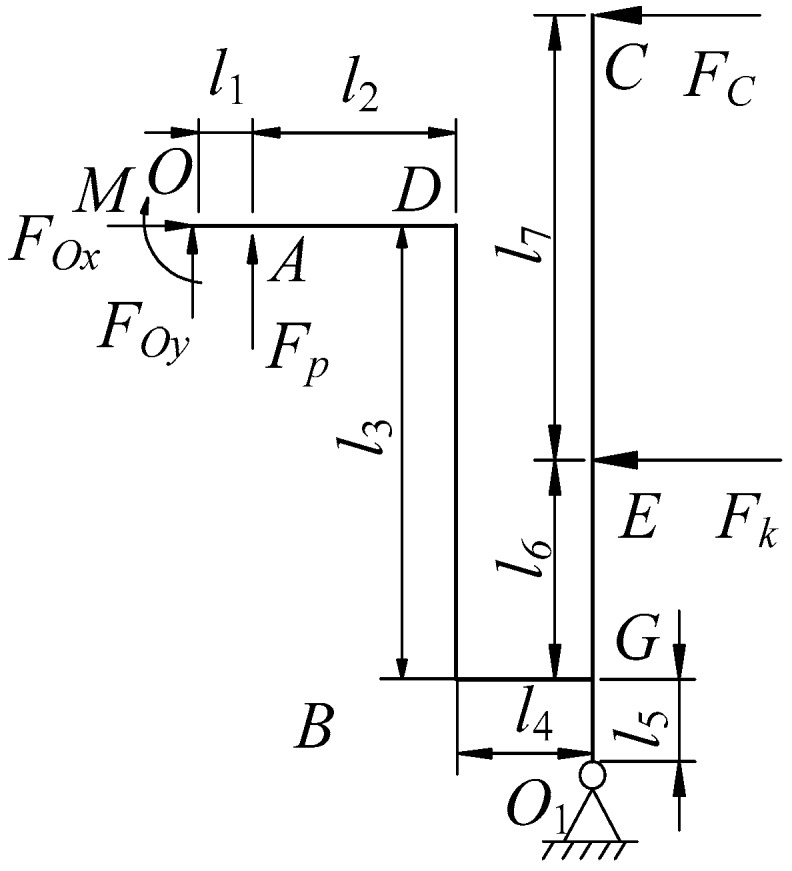
Forces of displacement amplification mechanism and swaying rod.

The driving source of the proposed micro piezoelectric precision drive system is derived from the two piezoelectric actuators. Thus, its exciting force is piezoelectric excitation. From the piezoelectric equations [26], the output force of the piezoelectric actuator can be obtained. The forces of the displacement amplification mechanism and the swaying rod are shown in Figure 2, where *F_c_* is the exciting force of the movable tooth system. From the force balance theorem, the force *F_c_* can be written as:

(1) $$\begin{array}{l} F_{C}=\frac{\left(F_{p}l_{1}-M_{O}\right)l_{5}-k\delta_{k}l_{3}(l_{5}+l_{6})}{l_{3}\left(l_{5}+l_{6}+l_{7}\right)}\\ \;=\frac{\left[\left(2l_{p}c_{33}s_{33}P+c_{33}d_{33}A_{p}U_{p}\right)l_{1}-2l_{p}M_{O}\right]l_{5}-2l_{p}k\delta_{k}l_{3}(l_{5}+l_{6})}{l_{3}\left(l_{5}+l_{6}+l_{7}\right)} \end{array},$$

where *c*_33_, *d*_33_, and *s*_33_ are elasticity modulus, elastic flexibility factor, and piezoelectric strain constant of the piezoelectric actuator, respectively; *P* is preload of piezoelectric actuator; *l_p_* and *A_p_* are the per piezoelectric pieces thickness and cross-sectional area of piezoelectric actuator, respectively; *U_p_* is the exciting voltage, $U_{p}=U_{p-p}\left[1+\sin\left(\omega t\right)\right]$, $U_{p-p}$ is the peak-peak value of voltage, *ω* is the exciting frequency; *Mo* is the torque of *O* point; *l_i_* is the length of each part, here, *l*_1_ = 6 mm, *l*_2_ = 11 mm, *l*_3_ = 35 mm, *l*_5_ = 6 mm, *l*_6_ = 16 mm, *l*_7_ = 44 mm; *k* and *δ_k_* are the stiffness and deformation of preloaded spring, respectively.

**Figure 3:**
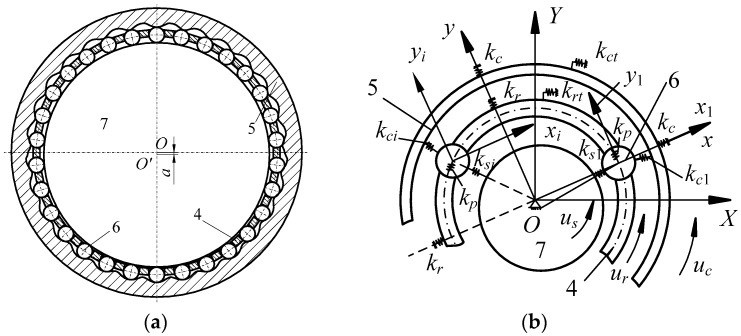
Dynamic model of the drive system. (**a**) Movable tooth structure; (**b**) model simplification.

Figure 3 shows the dynamic model of the drive system, where subscripts *s*, *c*, *r* and *p* represent the harmonic rod, central gear, rotor and movable tooth, respectively. Variables *x_j_*, *y_j_*, *u_j_* represent the *x* direction, *y* direction, and circumferential direction linear displacement, respectively. The nonlinear dynamic equations were built in [24], and the modeling process is simplified in this report. The nonlinear equations are used to analyze the nonlinear response characteristic. Substituting the nonlinear meshing stiffness into the dynamic model, the nonlinear dynamic equation of the drive system can be given as follows:

(2) $$M\ddot{q}+Kq=F+\Delta F+\Delta F_{e},$$

where ***M***, ***K***, and ***q*** are the mass matrix, stiffness matrix and generalized coordinate array, respectively. ***F***, Δ***F***, and Δ***F****_e_* are the outer force array, nonlinear meshing force increment array and exciting meshing force array, respectively (here, the force comes from the piezoelectric actuator).

The regularization dynamic equation can be written as:

(3) $$\Delta {\ddot{q}}_{N}+K_{N}\Delta q_{N}=\Delta F_{N}+\Delta F_{eN},$$

where ***K_N_*** is regular stiffness matrix, Δ***F_N_*** and Δ***F_eN_*** are the regular meshing force array and regular outer force array, respectively, and $\Delta F_{N}=A_{N}^{T}\Delta F=B_{k}u_{i}\varepsilon P_{N}^{T}$, Δ***q_N_*** is the regular coordinate array.

First, the nonlinear free vibration was investigated through the Linz Ted-Poincaré method. In this condition, the outer force array was zero, that is Δ***F_eN_*** = **0**.

Assuming a regular coordinate array Δ***q_N_***, natural frequency *ω_i_* can be expressed in the following forms:

(4) $$\left\{ \begin{array}{l} \Delta q_{N}=q_{N0}+\varepsilon q_{N1}+\varepsilon^{2}q_{N2}+\ldots \\ \omega_{i}^{2}=\omega_{0i}^{2}\left(1+\varepsilon \sigma_{1}+\varepsilon^{2}\sigma_{2}+\ldots \right) \end{array}\right.,$$

Substituting Equation (9) into Equation (8), with Δ***F_eN_*** = **0**, the nonlinear dynamic equation can be changed into a linear equation set as follows:

(5) $$\left\{ \begin{array}{l} {\ddot{q}}_{N0}+\omega_{0i}^{2}q_{N0}=0\\ {\ddot{q}}_{N1}+\omega_{0i}^{2}q_{N1}=-\sigma_{1}{\ddot{q}}_{N0}+P_{N}B_{k}u_{0i}\\ {\ddot{q}}_{N2}+\omega_{0i}^{2}q_{N2}=-\sigma_{1}{\ddot{q}}_{N1}-\sigma_{2}{\ddot{q}}_{N0}+\sigma_{1}P_{N}B_{k}u_{1i}\\ \ldots \ldots \end{array}\right.,$$

Solving the above equation set with a zero initial condition, the solutions of the zero-order equation, first-order equation and second-order equation can be expressed by:

(6) $$q_{N0}^{i}=A_{N0}^{i}\cos\left(\omega_{0i}t\right),$$

(7) $$\left\{ \begin{array}{l} q_{N1}^{1}=P_{N1}B_{k}\left(A_{N1}^{2}A_{N0}^{2}\frac{\cos\omega_{02}t-\cos\omega_{01}t}{\omega_{01}^{2}-\omega_{02}^{2}}+A_{N0}^{3}A_{N1}^{3}\frac{\cos\omega_{03}t-\cos\omega_{01}t}{\omega_{01}^{2}-\omega_{03}^{2}}\right)\\ q_{N1}^{2}=P_{N2}B_{k}\left(A_{N0}^{1}A_{N1}^{1}\frac{\cos\omega_{02}t-\cos\omega_{01}t}{\omega_{01}^{2}-\omega_{02}^{2}}+A_{N0}^{3}A_{N1}^{3}\frac{\cos\omega_{03}t-\cos\omega_{02}t}{\omega_{03}^{2}-\omega_{02}^{2}}\right)\\ q_{N1}^{3}=P_{N3}B_{k}\left(A_{N0}^{1}A_{N1}^{1}\frac{\cos\omega_{03}t-\cos\omega_{01}t}{\omega_{01}^{2}-\omega_{03}^{2}}+A_{N0}^{2}A_{N1}^{2}\frac{\cos\omega_{03}t-\cos\omega_{02}t}{\omega_{02}^{2}-\omega_{03}^{2}}\right) \end{array}\right.,$$

(8) $$\left\{ \begin{array}{l} q_{N2}^{1}=\frac{P_{N1}^{2}B_{k}^{3}A_{N1}^{1}\left(\cos\omega_{02}t-\cos\omega_{01}t\right)}{\omega_{02}^{2}-\omega_{01}^{2}}\left(\frac{P_{N2}A_{N0}^{1}A_{N1}^{1}A_{N1}^{2}}{\omega_{02}^{2}-\omega_{01}^{2}}+\frac{P_{N2}A_{N0}^{3}A_{N1}^{2}A_{N1}^{3}}{\omega_{02}^{2}-\omega_{03}^{2}}+\frac{P_{N3}A_{N0}^{2}A_{N1}^{2}A_{N1}^{3}}{\omega_{02}^{2}-\omega_{03}^{2}}\right)+\\ \;\frac{P_{N1}^{2}B_{k}^{3}A_{N1}^{1}\left(\cos\omega_{03}t-\cos\omega_{01}t\right)}{\omega_{03}^{2}-\omega_{01}^{2}}\left(\frac{P_{N2}A_{N0}^{3}A_{N1}^{2}A_{N1}^{3}}{\omega_{03}^{2}-\omega_{02}^{2}}+\frac{P_{N3}A_{N0}^{1}A_{N1}^{1}A_{N1}^{3}}{\omega_{03}^{2}-\omega_{01}^{2}}+\frac{P_{N3}A_{N0}^{2}A_{N1}^{2}A_{N1}^{3}}{\omega_{03}^{2}-\omega_{02}^{2}}\right)\\ q_{N2}^{2}=\frac{P_{N2}^{2}B_{k}^{3}A_{N1}^{2}\left(\cos\omega_{02}t-\cos\omega_{01}t\right)}{\omega_{02}^{2}-\omega_{01}^{2}}\left(\frac{P_{N1}A_{N0}^{2}A_{N1}^{2}A_{N1}^{1}}{\omega_{01}^{2}-\omega_{02}^{2}}+\frac{P_{N1}A_{N0}^{3}A_{N1}^{1}A_{N1}^{3}}{\omega_{01}^{2}-\omega_{03}^{2}}+\frac{P_{N3}A_{N0}^{1}A_{N1}^{1}A_{N1}^{3}}{\omega_{02}^{2}-\omega_{03}^{2}}\right)+\\ \;\frac{P_{N2}^{2}B_{k}^{3}A_{N1}^{2}\left(\cos\omega_{03}t-\cos\omega_{02}t\right)}{\omega_{03}^{2}-\omega_{02}^{2}}\left(\frac{P_{N1}A_{N0}^{3}A_{N1}^{1}A_{N1}^{3}}{\omega_{03}^{2}-\omega_{01}^{2}}+\frac{P_{N3}A_{N0}^{1}A_{N1}^{1}A_{N1}^{3}}{\omega_{03}^{2}-\omega_{02}^{2}}+\frac{P_{N3}A_{N0}^{2}A_{N1}^{2}A_{N1}^{3}}{\omega_{03}^{2}-\omega_{02}^{2}}\right)\\ q_{N2}^{3}=\frac{P_{N3}^{2}B_{k}^{3}A_{N1}^{3}\left(\cos\omega_{02}t-\cos\omega_{03}t\right)}{\omega_{02}^{2}-\omega_{03}^{2}}\left(\frac{P_{N1}A_{N0}^{2}A_{N1}^{2}A_{N1}^{1}}{\omega_{02}^{2}-\omega_{01}^{2}}+\frac{P_{N3}A_{N0}^{1}A_{N1}^{1}A_{N1}^{2}}{\omega_{02}^{2}-\omega_{01}^{2}}+\frac{P_{N3}A_{N0}^{3}A_{N1}^{2}A_{N1}^{3}}{\omega_{02}^{2}-\omega_{03}^{2}}\right)+\\ \;\frac{P_{N1}^{2}B_{k}^{3}A_{N1}^{3}\left(\cos\omega_{01}t-\cos\omega_{03}t\right)}{\omega_{01}^{2}-\omega_{03}^{2}}\left(\frac{P_{N1}A_{N0}^{3}A_{N1}^{2}A_{N1}^{1}}{\omega_{01}^{2}-\omega_{02}^{2}}+\frac{P_{N1}A_{N0}^{3}A_{N1}^{1}A_{N1}^{3}}{\omega_{01}^{2}-\omega_{03}^{2}}+\frac{P_{N3}A_{N0}^{1}A_{N1}^{2}A_{N1}^{1}}{\omega_{01}^{2}-\omega_{02}^{2}}\right) \end{array}\right.,$$

From $q_{N0}^{i}$, $q_{N1}^{i}$ and $q_{N2}^{i}$, the response displacement can be written as:

(9) $$q=A_{N}\left(q_{N0i}+\varepsilon q_{N1i}+\varepsilon^{2}q_{N2i}\right),$$

From the first-order equation and second-order equation, to eliminate the secular term, $\sigma_{1}^{i}$ can be achieved:

(10) $$\sigma_{1}^{i}=-\frac{A_{N1}^{i}P_{Ni}B_{k}}{\omega_{0i}^{2}},$$

(11) $$\left\{ \begin{array}{l} \sigma_{2}^{1}=\frac{P_{N1}B_{k}A_{N1}^{1}}{\omega_{01}^{2}A_{N0}^{1}}[\left(\frac{P_{N1}^{2}B_{k}^{2}A_{N1}^{1}}{\omega_{01}^{2}}-P_{N1}B_{k}\right)\left(\frac{A_{N0}^{2}A_{N1}^{2}}{\omega_{01}^{2}-\omega_{02}^{2}}+\frac{A_{N0}^{3}A_{N1}^{3}}{\omega_{01}^{2}-\omega_{03}^{2}}\right)+\\ \;\frac{P_{N1}B_{k}^{2}A_{N0}^{1}A_{N1}^{1}}{\omega_{01}^{2}}\left(\frac{P_{N2}}{\omega_{01}^{2}-\omega_{02}^{2}}+\frac{P_{N3}}{\omega_{01}^{2}-\omega_{03}^{2}}\right)]\\ \sigma_{2}^{2}=\frac{P_{N2}B_{k}A_{N1}^{2}}{\omega_{2}^{2}A_{N0}^{2}}[\left(\frac{P_{N2}^{2}B_{k}^{2}A_{N1}^{2}}{\omega_{02}^{2}}-P_{N2}B_{k}\right)\left(\frac{A_{N0}^{1}A_{N1}^{1}}{\omega_{02}^{2}-\omega_{01}^{2}}+\frac{A_{N0}^{3}A_{N1}^{3}}{\omega_{02}^{2}-\omega_{03}^{2}}\right)+\\ \;\frac{P_{N2}B_{k}^{2}A_{N0}^{2}A_{N1}^{2}}{\omega_{02}^{2}}\left(\frac{P_{N1}}{\omega_{02}^{2}-\omega_{01}^{2}}+\frac{P_{N3}}{\omega_{02}^{2}-\omega_{03}^{2}}\right)]\\ \sigma_{2}^{3}=\frac{P_{N3}B_{k}A_{N1}^{3}}{\omega_{03}^{2}A_{N0}^{3}}[\left(\frac{P_{N3}^{2}B_{k}^{2}A_{N1}^{3}}{\omega_{03}^{2}}-P_{N3}B_{k}\right)\left(\frac{A_{N0}^{1}A_{N1}^{1}}{\omega_{03}^{2}-\omega_{01}^{2}}+\frac{A_{N0}^{2}A_{N1}^{2}}{\omega_{03}^{2}-\omega_{02}^{2}}\right)+\\ \;\frac{P_{N3}B_{k}^{2}A_{N0}^{3}A_{N1}^{3}}{\omega_{03}^{2}}\left(\frac{P_{N1}}{\omega_{03}^{2}-\omega_{01}^{2}}+\frac{P_{N2}}{\omega_{03}^{2}-\omega_{02}^{2}}\right)] \end{array}\right.,$$

Thus, the nonlinear natural frequency can be written in the following form:

(12) $$\omega_{i}^{2}=\omega_{0i}^{2}\left(1+\varepsilon \sigma_{1}+\varepsilon^{2}\sigma_{2}\right),$$

In Equation (3), when Δ***F_eN_*** is not a zero value, the equation changes into a nonlinear forced vibration equation. Using the perturbation method, the nonlinear forced vibration equation can be solved. Firstly, transfer Equation (3) into a linear equation set as follows:

(13) $$\left\{ \begin{array}{l} {\ddot{q}}_{N0}+\omega_{0i}^{2}q_{N0}=0\\ {\ddot{q}}_{N1}+\omega_{0i}^{2}q_{N1}=-\sigma_{1}{\ddot{q}}_{N0}+P_{N}B_{k}u_{0i}+C_{k}\cos\left(\omega_{e}t\right)P_{eN}\\ {\ddot{q}}_{N2}+\omega_{0i}^{2}q_{N2}=-\sigma_{1}{\ddot{q}}_{N1}-\sigma_{2}{\ddot{q}}_{N0}+\sigma_{1}P_{N}Bu_{1i}+C_{k}\sigma_{1}\cos\left(\omega_{e}t\right)P_{eN}\\ \ldots \ldots \end{array}\right.,$$

where *C_k_* is constant parameter.

Solving the above linear equation set, the solution can be given as:

(14) $$q_{N0}^{i}=A_{N0}^{i}\cos\left(\omega_{0i}t\right),$$

(15) $$\left\{ \begin{array}{r} q_{N1}^{1}=P_{N1}B_{k}\left(A_{N1}^{2}A_{N0}^{2}\frac{\cos\omega_{02}t-\cos\omega_{01}t}{\omega_{01}^{2}-\omega_{02}^{2}}+A_{N0}^{3}A_{N1}^{3}\frac{\cos\omega_{03}t-\cos\omega_{01}t}{\omega_{01}^{2}-\omega_{03}^{2}}\right)\\ +\frac{C_{k}P_{eN1}\omega_{01}^{2}\left(\cos\omega_{e}t-\cos\omega_{01}t\right)}{\omega_{01}^{2}-\omega_{e}^{2}}\\ q_{N1}^{2}=P_{N2}B_{k}\left(A_{N0}^{1}A_{N1}^{1}\frac{\cos\omega_{02}t-\cos\omega_{01}t}{\omega_{01}^{2}-\omega_{02}^{2}}+A_{N0}^{3}A_{N1}^{3}\frac{\cos\omega_{03}t-\cos\omega_{02}t}{\omega_{03}^{2}-\omega_{02}^{2}}\right)\\ +\frac{C_{k}P_{eN2}\omega_{02}^{2}\left(\cos\omega_{e}t-\cos\omega_{02}t\right)}{\omega_{02}^{2}-\omega_{e}^{2}}\\ q_{N1}^{3}=P_{N3}B_{k}\left(A_{N0}^{1}A_{N1}^{1}\frac{\cos\omega_{03}t-\cos\omega_{01}t}{\omega_{01}^{2}-\omega_{03}^{2}}+A_{N0}^{2}A_{N1}^{2}\frac{\cos\omega_{03}t-\cos\omega_{02}t}{\omega_{02}^{2}-\omega_{03}^{2}}\right)\\ +\frac{C_{k}P_{eN3}\omega_{03}^{2}\left(\cos\omega_{e}t-\cos\omega_{03}t\right)}{\omega_{03}^{2}-\omega_{e}^{2}} \end{array}\right.,$$

(16) $$\left\{ \begin{array}{l} q_{N2}^{1}=\frac{P_{N1}B_{k}^{2}A_{N1}^{2}\left(\cos\omega_{02}t-\cos\omega_{01}t\right)}{\omega_{02}^{2}-\omega_{01}^{2}}\left[\frac{P_{N2}A_{N0}^{1}A_{N1}^{1}}{\omega_{02}^{2}-\omega_{01}^{2}}+\frac{\left(P_{N2}A_{N0}^{3}+P_{N3}A_{N0}^{2}\right)A_{N1}^{3}}{\omega_{02}^{2}-\omega_{03}^{2}}\right]+[\frac{P_{N3}A_{N0}^{1}A_{N1}^{1}}{\omega_{03}^{2}-\omega_{01}^{2}}+\\ \;\frac{\left(P_{N2}A_{N0}^{3}+P_{N3}A_{N0}^{2}\right)A_{N1}^{2}}{\omega_{03}^{2}-\omega_{02}^{2}}]\frac{P_{N1}B_{k}^{2}A_{N1}^{3}\left(\cos\omega_{03}t-\cos\omega_{01}t\right)}{\omega_{03}^{2}-\omega_{01}^{2}}+\frac{P_{N1}B_{k}P_{eN2}C_{k}\left(\cos\omega_{02}t-\cos\omega_{01}t\right)}{\left(\omega_{01}^{2}-\omega_{02}^{2}\right)\left(\omega_{02}^{2}-\omega_{e}^{2}\right)}+\\ \;\frac{P_{N1}B_{k}P_{eN3}C_{k}\left(\cos\omega_{03}t-\cos\omega_{01}t\right)}{\left(\omega_{03}^{2}-\omega_{01}^{2}\right)\left(\omega_{e}^{2}-\omega_{03}^{2}\right)}+\left(P_{eN1}+\frac{P_{N1}B_{k}P_{eN1}}{\omega_{02}^{2}-\omega_{e}^{2}}+\frac{P_{N1}B_{k}P_{eN3}}{\omega_{03}^{2}-\omega_{e}^{2}}\right)\frac{C_{k}\left(\cos\omega_{e}t-\cos\omega_{01}t\right)}{\omega_{01}^{2}-\omega_{e}^{2}}\\ q_{N2}^{2}=\frac{P_{N2}B_{k}^{2}A_{N1}^{1}\left(\cos\omega_{02}t-\cos\omega_{01}t\right)}{\omega_{02}^{2}-\omega_{01}^{2}}\left[\frac{P_{N1}A_{N0}^{2}A_{N1}^{2}}{\omega_{01}^{2}-\omega_{02}^{2}}+\frac{\left(P_{N1}A_{N0}^{3}+P_{N3}A_{N0}^{1}\right)A_{N1}^{3}}{\omega_{01}^{2}-\omega_{03}^{2}}\right]+[\frac{P_{N3}A_{N0}^{2}A_{N1}^{2}}{\omega_{03}^{2}-\omega_{02}^{2}}+\\ \;\frac{\left(P_{N1}A_{N0}^{3}+P_{N3}A_{N0}^{1}\right)A_{N1}^{1}}{\omega_{03}^{2}-\omega_{01}^{2}}]\frac{P_{N2}B_{k}^{2}A_{N1}^{3}\left(\cos\omega_{03}t-\cos\omega_{02}t\right)}{\omega_{03}^{2}-\omega_{02}^{2}}+\frac{P_{N2}B_{k}P_{eN1}C_{k}\left(\cos\omega_{02}t-\cos\omega_{01}t\right)}{\left(\omega_{01}^{2}-\omega_{02}^{2}\right)\left(\omega_{01}^{2}-\omega_{e}^{2}\right)}+\\ \;\frac{P_{N2}B_{k}P_{eN3}C_{k}\left(\cos\omega_{03}t-\cos\omega_{02}t\right)}{\left(\omega_{03}^{2}-\omega_{02}^{2}\right)\left(\omega_{e}^{2}-\omega_{03}^{2}\right)}+\left(P_{eN2}+\frac{P_{N2}B_{k}P_{eN1}}{\omega_{01}^{2}-\omega_{e}^{2}}+\frac{P_{N2}B_{k}P_{eN3}}{\omega_{03}^{2}-\omega_{e}^{2}}\right)\frac{C_{k}\left(\cos\omega_{e}t-\cos\omega_{02}t\right)}{\omega_{02}^{2}-\omega_{e}^{2}}\\ q_{N2}^{3}=\frac{P_{N3}B_{k}^{2}A_{N1}^{2}\left(\cos\omega_{02}t-\cos\omega_{03}t\right)}{\omega_{02}^{2}-\omega_{03}^{2}}\left[\frac{P_{N3}A_{N0}^{3}A_{N1}^{3}}{\omega_{02}^{2}-\omega_{03}^{2}}+\frac{\left(P_{N1}A_{N0}^{2}+P_{N3}A_{N0}^{1}\right)A_{N1}^{1}}{\omega_{02}^{2}-\omega_{01}^{2}}\right]+[\frac{P_{N1}A_{N0}^{3}A_{N1}^{3}}{\omega_{01}^{2}-\omega_{03}^{2}}+\\ \;\frac{\left(P_{N1}A_{N0}^{2}+P_{N3}A_{N0}^{1}\right)A_{N1}^{2}}{\omega_{01}^{2}-\omega_{03}^{2}}]\frac{P_{N3}B_{k}^{2}A_{N1}^{1}\left(\cos\omega_{03}t-\cos\omega_{01}t\right)}{\omega_{03}^{2}-\omega_{01}^{2}}+\frac{P_{N3}B_{k}P_{eN2}C_{k}\left(\cos\omega_{03}t-\cos\omega_{02}t\right)}{\left(\omega_{02}^{2}-\omega_{03}^{2}\right)\left(\omega_{02}^{2}-\omega_{e}^{2}\right)}+\\ \;\frac{P_{N3}B_{k}P_{eN1}C_{k}\left(\cos\omega_{03}t-\cos\omega_{01}t\right)}{\left(\omega_{01}^{2}-\omega_{03}^{2}\right)\left(\omega_{e}^{2}-\omega_{01}^{2}\right)}+\left(P_{eN3}+\frac{P_{N3}B_{k}P_{eN1}}{\omega_{01}^{2}-\omega_{e}^{2}}+\frac{P_{N3}B_{k}P_{eN2}}{\omega_{02}^{2}-\omega_{e}^{2}}\right)\frac{C_{k}\left(\cos\omega_{e}t-\cos\omega_{03}t\right)}{\omega_{03}^{2}-\omega_{e}^{2}} \end{array}\right.,$$

Substituting Equations (14)–(16) into Equation (9), the displacement of nonlinear forced vibration can be acquired.

## 4. Results and Discuss 

### 4.1. Nonlinear Free Vibration Analysis

**Figure 4:**
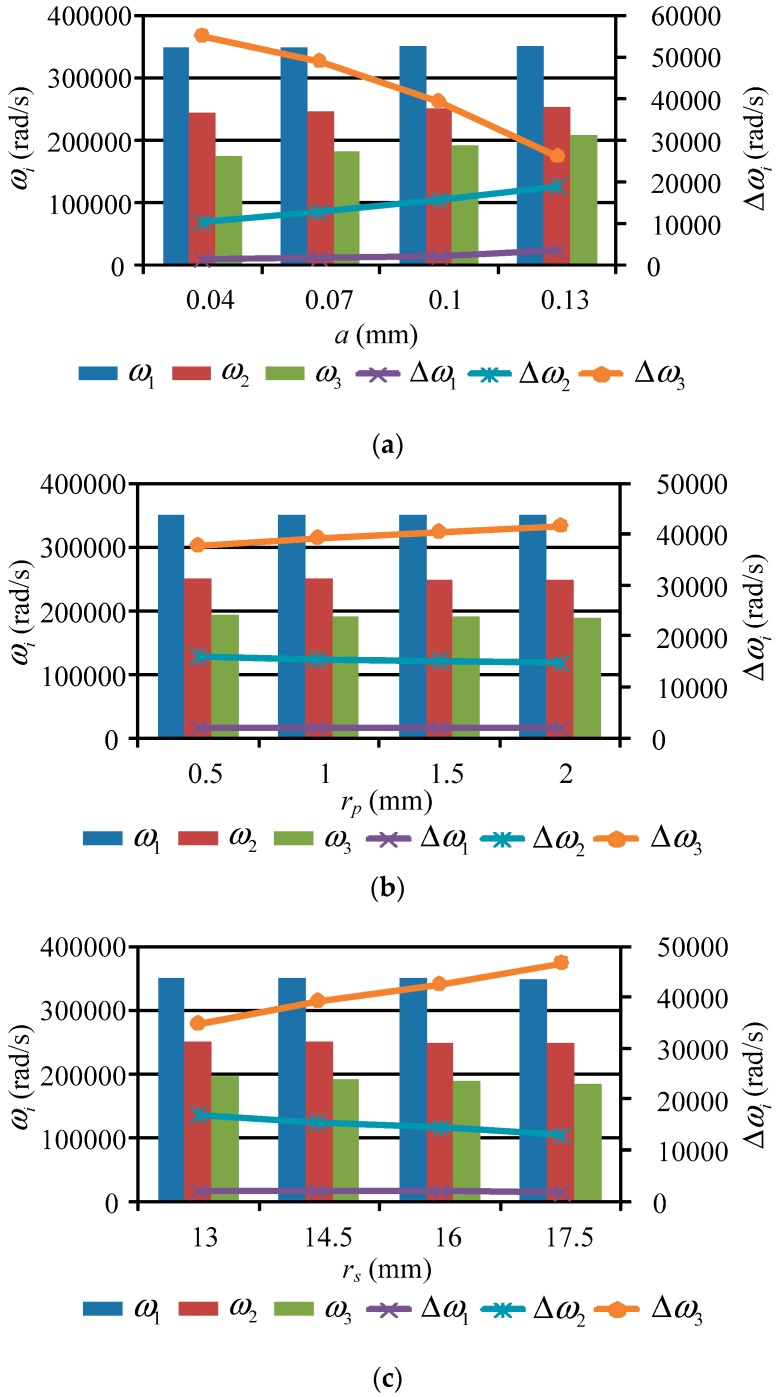
Effect of the parameters on nonlinear natural frequencies. (**a**) Harmonic offset *a* change; (**b**) movable tooth radius *r_p_* changes; (**c**) harmonic plate radius *r_s_*.

Table 1 presents the system parameters of the drive system. By substituting the parameters into the frequency equation, Equation (12), the nonlinear natural frequencies of the proposed piezoelectric precision drive system were found (see Table 2). Meanwhile, by selecting the harmonic offset *a*, movable tooth radius *r_p_*, and harmonic plate radius *r_s_* as research parameters, the effect of the parameters on nonlinear natural frequencies was investigated, as presented in Figure 4. In Table 2 and Figure 4, *ω_i_* and Δ*ω_i_* represent nonlinear natural frequencies and the frequency difference between nonlinear and linear frequencies. From Table 2 and Figure 4, it can be observed that:

(1) Under the influence of the nonlinear effect, the first-order and second-order frequencies are larger than their linear natural frequencies, whereas the third-order frequency is smaller than its linear natural frequency. As the frequency order increases, the frequency difference between the nonlinear and linear frequencies grows. When the number of meshing movable teeth is 16, the frequency difference is less than that with 15 meshing teeth. Hence, the less meshing teeth there are, the stronger the nonlinear phenomenon.

(2) As the harmonic offset *a* grows, the nonlinear frequency of each order increases. This is because the meshing forces between the movable tooth and each element become larger with an increase in *a*, resulting in growth of their meshing stiffness. For the first-order and second-order conditions, the frequency difference Δ*ω_i_* increases when *a* increases, while it decreases for the third-order condition. Thus, the nonlinear phenomenon of the first-order and second-order frequencies strengthens with the increase of harmonic offset *a*.

(3) The variation rules of nonlinear frequencies *ω_i_* and frequency difference Δ*ω_i_* are similar to the change in movable tooth radius *r_p_* and harmonic plate radius *r_s_*. As *r_p_* and *r_s_* grow, the nonlinear frequencies decrease, and the reason for this lies in the decreasing meshing stiffness. The frequency differences of the first-order and second-order conditions are reduced with the increase in *r_p_* and *r_s_*, while the value is increased for the third-order condition. Hence, the nonlinear intensities of the first-order and second-order conditions are directly proportional to changes in *r_p_* and *r_s_*, while that of the third-order condition is inversely proportional. 

**Figure 5:**
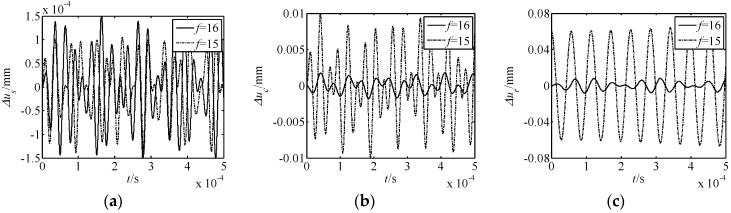
Effect of the meshing movable teeth *f* on nonlinear free vibration responses. (**a**) Δ*u_s_* response; (**b**) Δ*u_c_* response; (**c**) Δ*u_r_* response.

**Figure 6:**
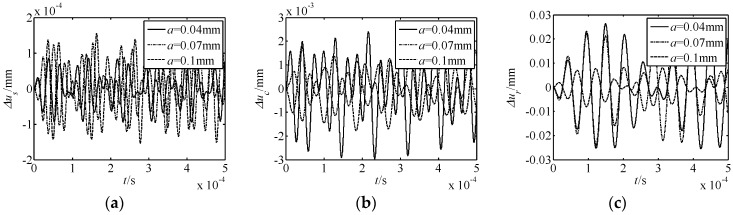
Effect of the harmonic offset *a* on nonlinear free vibration responses. (**a**) Δ*u_s_* response; (**b**) Δ*u_c_* response; (**c**) Δ*u_r_* response.

**Figure 7:**
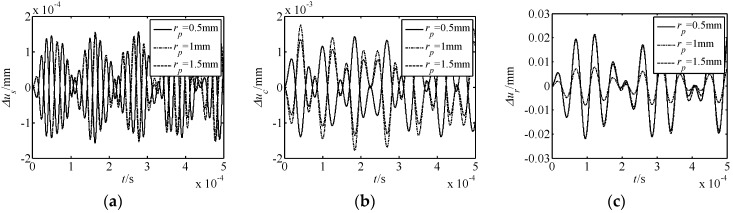
Effect of the movable tooth radius *r_p_* on nonlinear free vibration responses. (**a**) Δ*u_s_* response; (**b**) Δ*u_c_* response; (**c**) Δ*u_r_* response.

**Figure 8:**
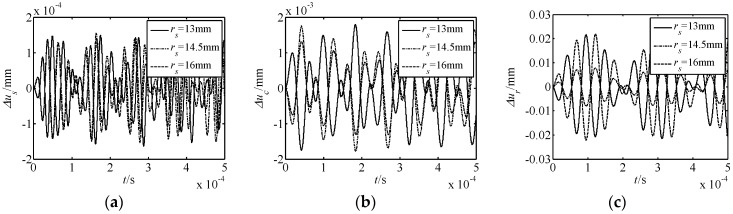
Effect of the harmonic plate radius *r_s_* on nonlinear free vibration responses. (**a**) Δ*u_s_* response; (**b**) Δ*u_c_* response; (**c**) Δ*u_r_* response.

The influence of parameters on nonlinear free vibration responses was also investigated. For this purpose, the parameters of the number of meshing movable teeth *f*, harmonic offset *a*, movable tooth radius *r_p_*, and harmonic plate radius *r_s_* were selected to be investigated. The acquired results are shown in Figure 5, Figure 6, Figure 7 and Figure 8. In the figures, Δ*u_s_*, Δ*u_c_*, and Δ*u_r_* represent the torsional responses of the harmonic rod, central gear, and rotor. In Figure 5, Figure 6, Figure 7 and Figure 8, it can be observed that:

(1) The number of meshing movable teeth *f* has an important influence on the nonlinear displacement responses of the novel drive system. When the number of meshing movable teeth *f* changes from 16 to 15, the amplitude of Δ*u_s_* reduces by a third, whereas the values of Δ*u_c_* and Δ*u_r_* increase 5- and 6-fold. Hence, with less meshing movable teeth, the nonlinear torsional vibration of the harmonic rod weakens, and the nonlinear torsional vibration of the central gear and rotor increases.

(2) As the parameter *a* increases, the amplitude of Δ*u_s_* increases, while that of Δ*u_c_* and Δ*u_r_* decreases. Thus, the nonlinear vibration of the harmonic rod enhances as *a* increases, and those of the central gear and rotor weaken. From the large variation of Δ*u_c_* and Δ*u_r_*, the influence of the nonlinear effect on the central gear and rotor is larger than that of the harmonic rod.

(3) The effect laws of movable tooth radius *r_p_* and harmonic plate radius *r_s_* on the displacement response are similar. As *r_p_* and *r_s_* increase, the amplitude of Δ*u_s_* decreases, the amplitude of Δ*u_c_* firstly increases and then decreases, and that of Δ*u_c_* firstly decreases and then increases. In addition, the range of Δ*u_s_* is smaller to that of Δ*u_c_* and Δ*u_r_*.

In summary, under all conditions, the effect of the number of meshing movable teeth *f* on the displacement response is the most obvious. Further, for the nonlinear torsional vibration of each element, the rotor is most affected by the nonlinear phenomenon.

### 4.2. Nonlinear Forced Vibration Analysis

**Figure 9:**
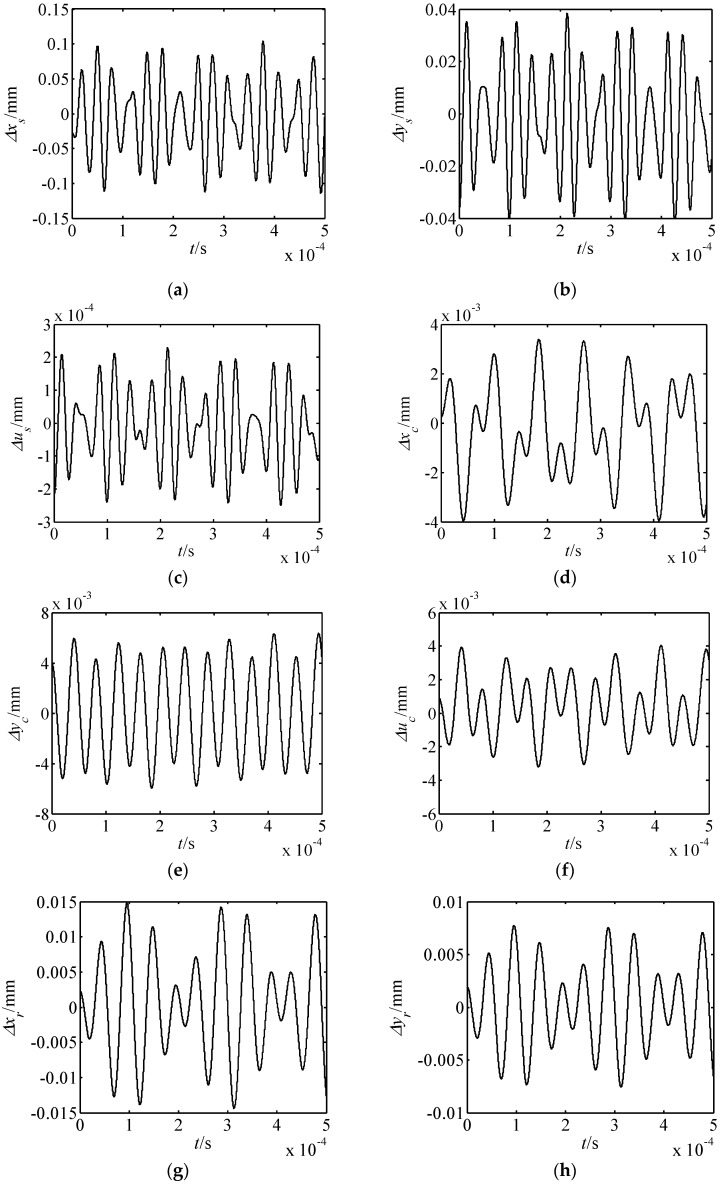

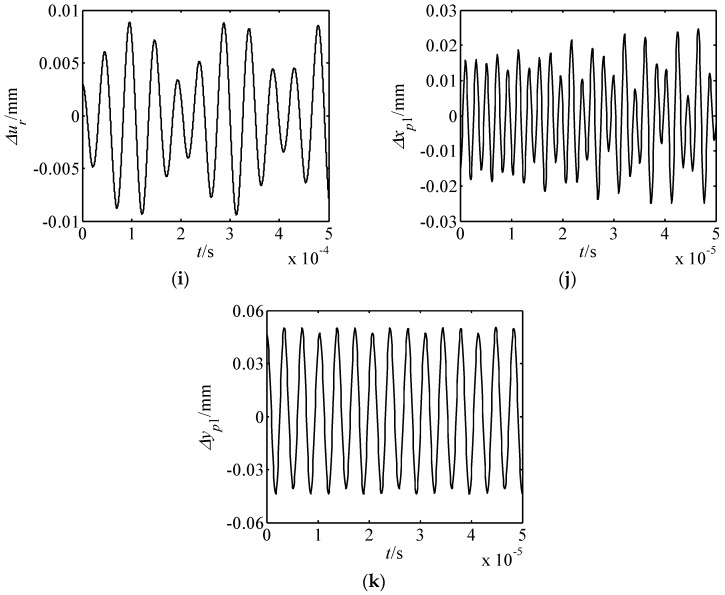
Displacement responses of the drive system far from resonance. (**a**) Δ*x_s_* response; (**b**) Δ*y_s_* response; (**c**) Δ*u_s_* response; (**d**) Δ*x_c_* response; (**e**) Δ*y_c_* response; (**f**) Δ*u_c_* response; (**g**) Δ*x_r_* response; (**h**) Δ*y_r_* response; (**i**) Δ*u_r_* response; (**j**) Δ*x_p_*_1_ response; (**k**) Δ*y_p_*_1_ response.

**Figure 10:**
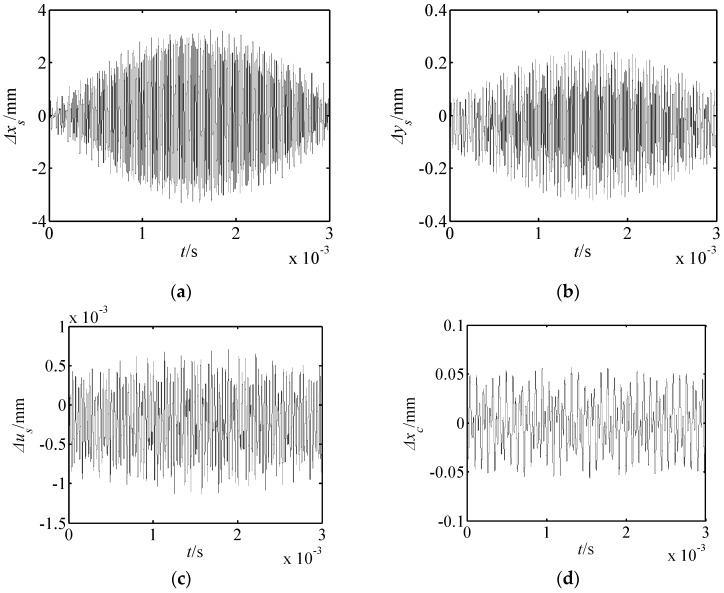

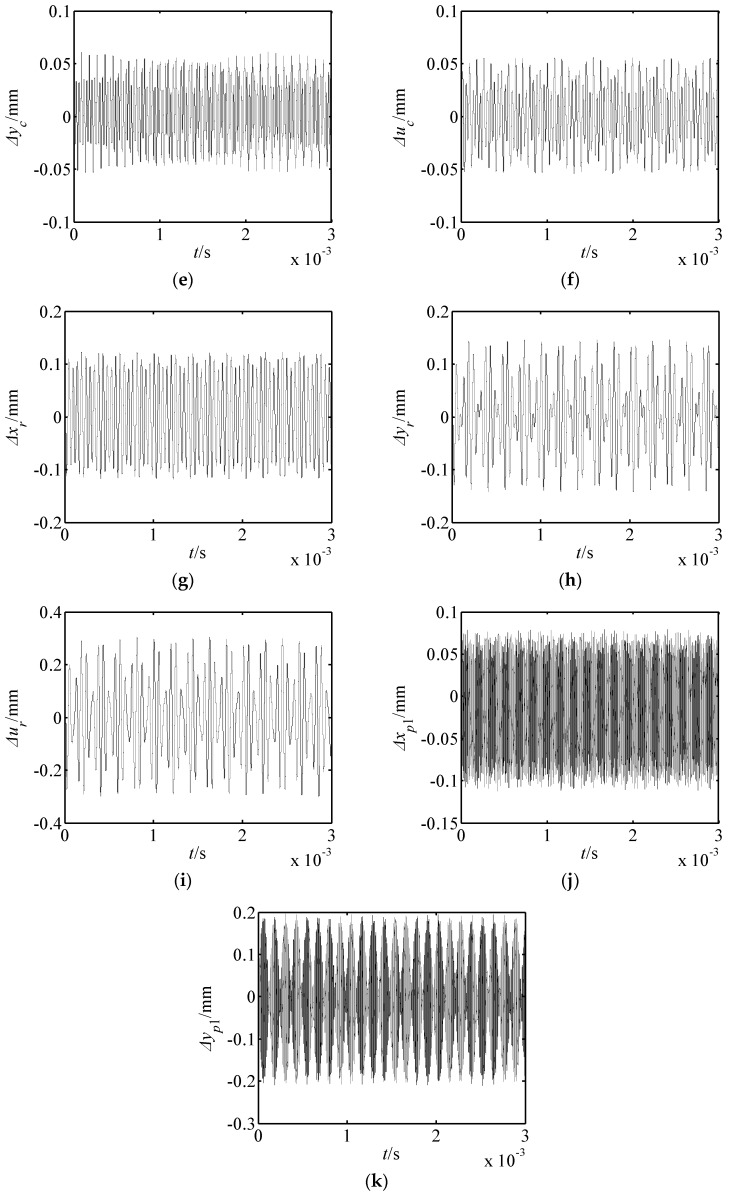
Displacement responses of the drive system close to resonance. (**a**) Δ*x_s_* response; (**b**) Δ*y_s_* response; (**c**) Δ*u_s_* response; (**d**) Δ*x_c_* response; (**e**) Δ*y_c_* response; (**f**) Δ*u_c_* response; (**g**) Δ*x_r_* response; (**h**) Δ*y_r_* response; (**i**) Δ*u_r_* response; (**j**) Δ*x_p_*_1_ response; (**k**) Δ*y_p_*_1_ response.

From Equations (14)–(16) and Equation (9), the nonlinear forced vibration responses of the proposed drive system can be determined. When the outer exciting frequency (*ω_e_* = 1000 rad/s) was far from the resonance frequency, this effected the displacement responses of the drive system, as shown in Figure 9. When the outer exciting frequency was close to the first-order resonance frequency, this effected the close resonance responses, which are presented in Figure 10. From Figure 9 and Figure 10, it is known that:

(1) When the outer exciting frequency is far from the resonance frequency, the amplitude of each element is quite small.

(2) The translational vibration displacements of the harmonic rod and movable teeth are larger than those of other elements. In addition, these characteristics are consistent with the motion feature of the proposed piezoelectric precision drive system. When the drive system operates, the swaying movement of harmonic rod occurs. Then, the harmonic forces push the movable teeth to generate two-dimensional motion.

(3) When the outer exciting frequency is close to the resonance frequency, the response amplitudes in the *x_s_* and *y_s_* directions are obviously larger than that far from the resonance frequency. At this point, resonances in the *x_s_* and *y_s_* directions occur, and the resonance in the *x_s_* direction is much more severe. Hence, the vibration mode is translational vibration of the harmonic rod for the first-order resonance.

### 4.3. Simulation Verification Analysis

**Figure 11:**
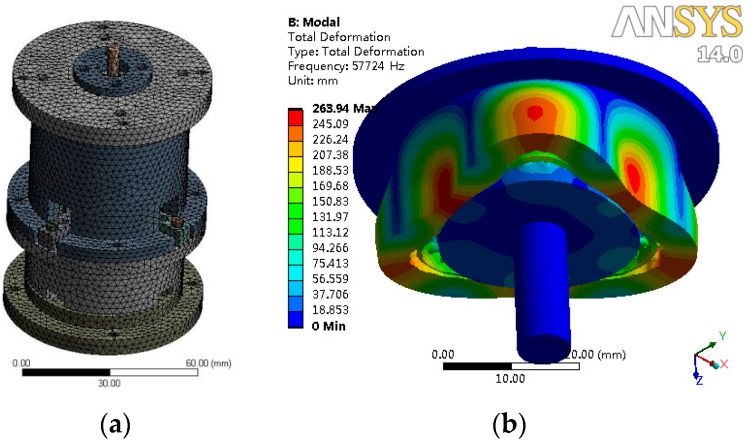
Finite elements method (FEM) simulation of nonlinear frequencies. (**a**) Mesh generation; (**b**) first-order vibration mode.

To verify the correctness of the theoretical model, a finite element method (FEM) software, ANSYS, was used to simulate the nonlinear frequencies of the system. When building the 3D model, the number of meshing movable teeth was set to 16. The FEM model and first-order vibration mode are shown in Figure 11. The theoretical and simulated nonlinear natural frequencies are presented in Table 3. 

The results show that the first-order and second-order simulated frequencies were larger than their theoretical values, while the third-order simulation frequency was smaller than its theoretical value. As the frequency order increased, the errors between the theoretical and simulated frequencies were reduced. The largest error was less than 4%.

## 5. Conclusions

In this study, a micro piezoelectric precision drive system was proposed, and its operating principle was presented. Considering the nonlinear effect of the change in the number of meshing movable teeth, the nonlinear dynamic model and equations of the proposed drive system were established. Using the Linz Ted-Poincaré and perturbation methods, the solutions of the nonlinear responses were calculated. The results indicate that:

(1) The nonlinear intensity of the drive system is inversely proportional to the number of meshing movable teeth.

(2) Regarding the nonlinear torsional vibration of each element, the rotor is most affected by the nonlinear phenomenon.

(3) For the first-order resonance, translational vibration is obvious in the system.

These results can be used for optimizing the structure and improving the dynamic properties of the proposed micro piezoelectric precision drive system.

## Figures and Tables

**Table 1:** Parameters of the drive system.

Parameters	Rotor	Central Gear	Harmonic Rod	Movable Tooth
*m_j_* (Kg)	1.31 × 10^−^^2^	5.64 × 10^−2^	2.59 × 10^−2^	3.30 × 10^−5^
*I_j_* (Kg)	9.34 × 10^−3^	8.41 × 10^−2^	1.30 × 10^−2^	1.32 × 10^−5^
*r_j_* (mm)	31.6	33.2	29	2

**Table 2:** Nonlinear natural frequencies of the drive system.

Number of Meshing Teeth	Frequencies (rad/s)	*ω*_01_	*ω*_02_	*ω*_03_
16	*ω*_0*i*_	347,066	233,801	230,295
	*ω_i_*	349,023	249,219	191,179
	Δ*ω_i_*	1957	15,418	39,116
	Δ*ω_i_*/*ω*_0*i*_ × 100	0.56	6.59	16.99
15	*ω*_0*i*_	346,874	235,535	229,473
	*ω_i_*	336,116	269,738	168,614
	Δ*ω_i_*	10,758	34,202	60,858
	Δ*ω_i_*/*ω*_0*i*_ × 100	3.10	14.52	26.52

**Table 3:** Comparison of nonlinear frequency simulation results.

Order	*ω*_1_	*ω*_2_	*ω*_3_
Theoretical value (rad/s)	349,023	249,219	191,179
Simulation value (rad/s)	362,690	254,236	190,217
Error (%)	3.92	2.01	0.50
